# Non-suicidal self-injury and bulimia: the role of emotion dysregulation and body dissatisfaction

**DOI:** 10.1007/s40519-019-00741-5

**Published:** 2019-07-10

**Authors:** Lindsey Hovrud, Raluca Simons, Jeffrey Simons, John Korkow

**Affiliations:** University of South Dakota, 414 E Clark St, South Dakota Union Building, Vermillion, SD 57069 USA

**Keywords:** Body dissatisfaction, Bulimia, Non-suicidal self-injury, Distress tolerance, Negative urgency, Emotion dysregulation

## Abstract

**Purpose:**

Risk factors of negative affect, body dissatisfaction, distress tolerance, and negative urgency are independently associated with bulimia symptoms and non-suicidal self-injury (NSSI). However, relationships of these risk factors within comorbid presentations are not fully understood. The current study examined specific roles of these risk factors within this relationship.

**Methods:**

An at-risk community sample of young adults (*N* = 429) completed an online survey of negative affect, body dissatisfaction, distress tolerance, negative urgency, bulimia symptoms, and NSSI.

**Results:**

A hypothesized path model was a good fit to the data. Results indicated direct paths from body dissatisfaction, negative urgency, and distress tolerance to bulimia symptoms. Negative urgency, distress tolerance, and bulimia symptoms were directly associated with NSSI. Consistent with hypotheses, distress tolerance and negative urgency acted as vulnerability factors, increasing the strength of associations between bulimia symptoms and NSSI. Distress tolerance also strengthened associations between negative urgency and NSSI. In addition to the direct effect, negative urgency was indirectly associated with NSSI via body dissatisfaction bulimia. Hypothesized indirect effects through distress tolerance were not supported.

**Conclusions:**

Results support etiological models of bulimia and NSSI, and suggest deficits in emotion regulation strengthen risk of comorbid presentations. Furthermore, individuals with greater impulsivity and difficulty tolerating distress are at increased risk of engaging in both bulimia behaviors and NSSI, providing targets for clinical intervention.

**Level of evidence:**

Level V, cross-sectional descriptive study.

## Introduction

A well-established relationship exists between non-suicidal self-injury (NSSI) and bulimia, where prevalence rates of self-harm among individuals with bulimia behaviors range from 26 to 61% [1, 2]. Regarding temporal precedence of this relationship, a prospective study suggests bulimia symptoms predict engagement in self-harm behaviors [2]. Additional impairments are observed among individuals with comorbid presentations, including increased suicide attempts, substance use, and other externalizing behaviors [3, 4]. These impairments contribute to increased clinical costs and medical burden [3], and understate the importance of understanding mechanisms that contribute to the development of comorbid presentations of bulimia symptoms and NSSI.

Regarding prevalence, engagement in bulimia behaviors is common among college-aged populations, with up to 16% and 9% of women and men, respectively, considered at risk of developing a formal diagnosis [5, 6]. While self-harm behaviors are often studied among pre-adolescents, high prevalence rates of NSSI are observed among college-aged populations, ranging from 12 to 38% [3, 7, 8]. These rates suggest college-aged populations are an at-risk group for comorbid bulimia and NSSI behaviors.

The experiential avoidance model [9] suggests engagement in self-injury facilitates temporary relief and avoidance of undesired emotional states. This model highlights the role of negative affect across dysregulated behaviors, and may account for elevated rates of NSSI among individuals with bulimia behaviors [10, 11]. Specifically, those with bulimia behaviors display tendencies towards affective avoidance [12, 13]. Thus, the experiential avoidance model provides a theoretical framework to conceptualize the role of negative affect within the comorbidity of NSSI and bulimia symptoms.

Negative affect is likely necessary but not sufficient in fully accounting for this comorbidity. Thus, vulnerability factors that facilitate experiential avoidance should be examined. Distress tolerance, defined as the capacity to withstand negative states [14], is a risk factor underlying engagement in NSSI [7, 15] and bulimia pathology [16, 17]. Similarly, negative urgency, defined as the tendency to engage in impulsive behaviors under negative affect [18], has been linked to NSSI [7, 19] and has uniquely predicted bingeing and purging episodes [20, 21]. This trait impulsivity, observed in engagement of bulimia behaviors, has also predicted the presence of NSSI across clinical [22] and college populations [2]. Distress tolerance and negative urgency have also been conceptualized as proximal and distal risk factors, respectively, within the experiential avoidance model [3]. Together, these findings suggest deficits in the ability to withstand aversive states and the tendency to act rashly under these states may contribute to the relationship of bulimia and NSSI.

Finally, greater body dissatisfaction has been observed among those reporting NSSI compared to non-clinical controls [23], and has mediated the relationship between negative affect and NSSI [24]. This is also considered a proximal risk factor within the experiential avoidance model of NSSI [3]. Similarly, individuals diagnosed with an eating disorder with a history of NSSI endorse higher body dissatisfaction compared to non-eating disorder individuals with NSSI [1, 24]. These robust findings suggest body dissatisfaction is a risk factor for this comorbidity, consistent with the hypothesized theoretical framework.

Body dissatisfaction, negative affect, distress tolerance, and negative urgency have not yet been examined concurrently within an at-risk population. The current study tested a moderated-mediation path model. It was expected that direct effects from bulimia would vary given the level of moderator. We hypothesized the following: (1) body dissatisfaction and bulimia would mediate the relationship between negative affect and NSSI, (2) bulimia would mediate the relationship between body dissatisfaction and NSSI, (3) distress tolerance would be associated with NSSI directly, and indirectly, via bulimia, (4) negative urgency would be associated with NSSI directly, and indirectly, via bulimia, (5) distress tolerance and negative urgency would moderate the relationship between bulimia and NSSI, and (6) negative urgency would moderate the relationship between distress tolerance and NSSI. Given positive associations with female gender and bulimia symptoms [21, 25], gender was included as a covariate.

## Methods

### Participants

The full sample included 488 young adults, ranging from 18 to 25 years old, recruited via a Midwestern university research system (*N* = 241) and Amazon MTurk (*N* = 247). The full sample was 37.7% male, 59.6% female, 1.2% identified as agender, genderfluid, or gender nonbinary, and 1.5% did not respond. Participants who did not identify as male or female completed a free response item regarding gender. Approximately 58% were White/Caucasian, 4% were Black/African American, 4% were Native American/Alaskan Native, 26% Asian/Asian American, 1% were Native Hawaiian/Pacific Island, 4% were Multiracial, 2% identified as Other, and 1% did not respond. Approximately 11% identified as Latino/Hispanic. Age range was assessed through MTurk (age 18–25), but did not capture specific ages of participants. Thus, age-specific descriptive information was obtained only for university-recruited participants (Table 1).

**Table 1:** Descriptive statistics

Variable	*N*	*M* (*SD*)	Range	Skewness	Kurtosis
Gender	423	–	160 (M), 263 (F)	–	–
Age	229	19.43 (1.43)	18–24	1.26	4.51
EDI—bulimia	425	18.99 (7.79)	5–43	0.54	2.49
EDI—body dissatisfaction	425	33.25 (10.70)	7–60	− 0.02	2.75
NSSI (yes/no)	424	–	218 (Y), 206 (N)	–	–
Negative affect	422	2.63 (0.90)	1–5	0.24	2.28
DTS	424	3.03 (0.94)	1–5	− 0.16	2.27
Negative urgency	423	2.37 (0.70)	1–3.92	− 0.17	2.24

*N*s differ due to missing data. *N* differs in age due to inclusion criteria for MTurk participants. EDI = eating disorder inventory, NSSI = non-suicidal self-injury, DTS = distress tolerance. Gender-coded 1 (male) and 0 (female). NSSI is dichotomous (positive history = 1, no history = 0)

### Measures

#### Screening questionnaire

Participants recruited through Amazon MTurk received the How I Deal with Stress measure (HIDS) [26] to screen for engagement of self-injury. The HIDS is a 24-item, self-report questionnaire that measures strategies used to cope with stress. Responses are rated on a four-point scale where participants are asked to indicate to what extent they have used each strategy to cope with stress. Eligibility for participants recruited via the university research program was contingent on age, and thus did not receive the HIDS.

#### Bulimia and body dissatisfaction

Bulimia symptoms and body dissatisfaction were measured by the Eating Disorder Inventory-3rd Edition (EDI-3) [27]. The EDI-3 is a self-report inventory that measures the main cognitive and behavioral features of dysregulated eating pathology with sound specificity and sensitivity related to problematic symptoms [28, 29]. Only the subscales of bulimia and body dissatisfaction were used. Responses are rated on a six-point scale, where greater, summed values indicate more severe symptomology. The EDI-3 has demonstrated good validity and internal consistency (.81–.93) among college populations [30], and demonstrated good consistency in the current sample (α = .88) and (α = 087), respectively.

#### NSSI

Self-injury was measured by the Deliberate Self-Harm Inventory (DSHI) [31]. The DSHI is comprised of 17 questions, where each item is followed by five questions to assess onset, frequency, severity, and duration of each self-harm behavior. A dichotomous (yes/no) variable was created, where endorsement of any self-harm indicated lifetime-NSSI. The dichotomous outcome has demonstrated high internal consistency (α = .82) [31], and demonstrated good consistency in the current sample (α = .86).

#### Negative affect

Negative affect was measured by the Positive Affect Negative Affect Schedule (PANAS) [32]. This 20-item, self-report measure captures the extent to which participants experience affective states. Participants rated their responses “on average.” Mean scores for negative affect items were obtained with greater scores indicating greater levels of negative affect. The PANAS has demonstrated good internal consistency among college populations (α = .87) [33], and displayed good consistency among the current sample (α = .90).

#### Distress tolerance

Distress tolerance was measured by the Distress Tolerance Scale (DTS) [14]. This 15-item, self-report measure, assesses the extent one can withstand distressing psychological states. The DTS includes four subscales (tolerance, appraisal, absorption, and regulation), which have demonstrated good internal consistency (α = .72, .83, .78, and .72, respectively) [34]. An overall DTS score was obtained by calculating the mean of the subscales, where high scores indicated greater tolerance for distress. This measure displayed good consistency among the current sample (α = .94).

#### Negative urgency

The tendency to act rashly when experiencing negative affect was measured using the negative urgency subscale of the UPPS Impulsive Behavior Scale [18]. This 45-item, self-report measure assesses impulsivity across dimensions of the Five Factor Model of personality. The UPPS has high internal consistency (α > .87) within college populations [27], and demonstrated good consistency in the current sample (α = .92).

#### Procedure

Participants were recruited through an online, Midwestern university research system and via Amazon MTurk. The study was described as a survey of personal preferences and emotion. No advertising outside of the posted survey through the online databases was used for this study. Participants between the ages of 18 and 25 were eligible to participate. Eligibility through Amazon MTurk was also contingent on at least one occurrence of NSSI as a method to deal with stress. Participants provided informed consent and procedures were approved by the university’s review board. Anonymous surveys were completed online. Participants were awarded course credit or monetary payment ($2.10 per survey via Amazon MTurk) as compensation. Participants recruited through Amazon MTurk who were not eligible for participation received $0.10 for completion of the HIDS screening survey.

### Statistical analyses

The data sets analyzed for the current study are available from the corresponding author on reasonable request. Descriptive analyses were conducted using Stata 14 [35] and the hypothesized path model was tested using Mplus 7.3 [36]. First, missing data and reliability were examined through validity questions embedded across both recruitment methods. Cases were removed due to incomplete surveys or inconsistent/unreliable responses, improving psychometrics from recruitment methods. Participants who identified as agender (*N* = 1), genderfluid (*N* = 1), or gender nonbinary (*N* = 3) were removed from analysis. Thus, the analyzed sample ranged from *N* = 422–425, dependent on the observed variable. Observed variables were examined for outliers and univariate normality was examined using histograms, skewness, and kurtosis values (Table 1). Assumptions of logistic regression were examined. The correlation matrix (Table 2) and tolerance values suggest absence of problematic collinearity [37].

**Table 2:** Correlations between observed variables

	1.	2.	3.	4.	5.	6.	7.
1. Gender	–						
2. NSSI	0.04	–					
3. EDI—bulimia	0.13**	0.34***	–				
4. EDI—body dissatisfaction	− 0.17***	0.21***	0.44***	–			
5. Distress tolerance	− 0.11*	− 0.47***	− 0.37***	− 0.28***	–		
6. Negative urgency	0.09	0.46***	0.52***	0.32***	− 0.59***	–	
7. Negative affect	0.13**	0.35***	0.38***	0.25***	− 0.55***	0.51***	–

EDI = eating disorder inventory, NSSI = non-suicidal self-injury; gender coded 1 (male) and 0 (female). NSSI is dichotomous (positive history = 1, no history = 0)

*p < 0.05, **p < 0.01, ***p < 0.001

## Results

### Descriptive and bivariate statistics

Approximately 25% (*N* = 56) of the university sample endorsed at least one incident of lifetime-NSSI, comparable to prevalence rates among college populations [7]. Despite endorsement assessed by the HIDS inclusion criteria measure, 18% of the final MTurk sample did not endorse self-harm, as measured by the DSHI. Of the total sample, approximately 52% (*N* = 219) endorsed at least one incident of lifetime-NSSI. Forty-nine percent of women and 51% of men reported histories of NSSI within the full sample. Likelihood of NSSI engagement did not differ as a function of gender (*Χ*^2^ [1, *N* = 429] = 0.64, *p* = .423).

### Path analysis

The hypothesized path model was tested and conditional effects were examined. The full model included gender, negative affect, negative urgency, distress tolerance, and interactions of bulimia-negative urgency, bulimia–distress tolerance, and distress tolerance-negative urgency as exogenous variables. NSSI was a dichotomous outcome and modeled with a logit link; thus, the weighted least squares mean values and variance (WLSMV)-adjusted estimator were used in the analysis. Negative affect had a direct effect on body dissatisfaction, which had a direct effect on bulimia. Negative affect also had an indirect effect on NSSI via body dissatisfaction and bulimia. Negative urgency and distress tolerance had direct effects on bulimia and NSSI, and indirect effects on NSSI via bulimia. Lastly, interactions of bulimia-distress tolerance, bulimia-negative urgency, and distress tolerance-negative urgency had direct effects on NSSI. Gender was included as a covariate and all exogenous variables were allowed to freely correlate. The endogenous bulimia disturbance term was allowed to co-vary with the bulimia-negative urgency and bulimia–distress tolerance interactions [38].

**Fig. 1:**
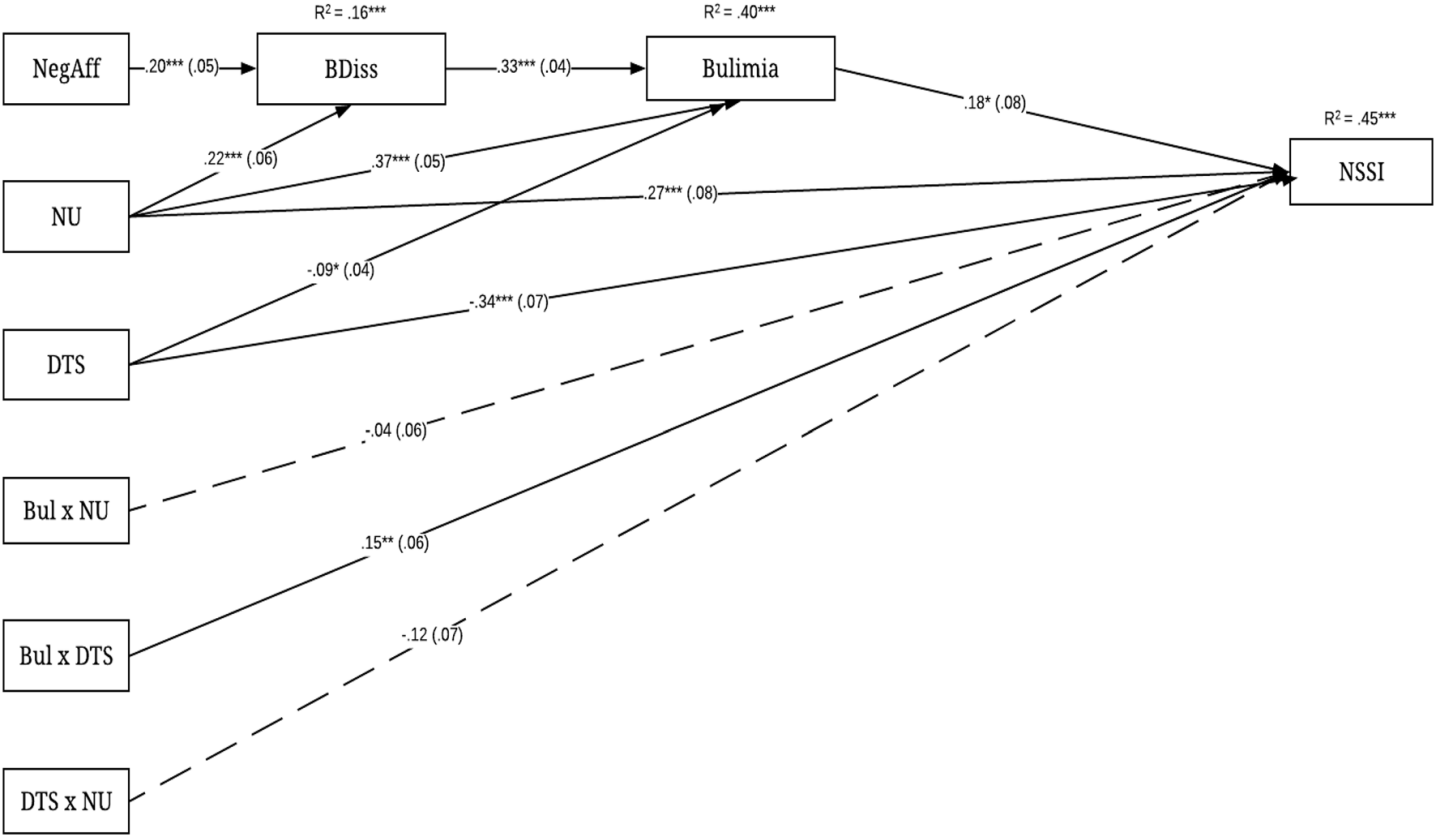
Final model of emotion dysregulation and body dissatisfaction on NSSI. NegAff = negative affect; BDiss = body dissatisfaction; DTS = distress tolerance; NU = negative urgency; BulxDTS = distress tolerance and bulimia interaction; DTSxNU = distress tolerance and negative urgency interaction; BulxNU = bulimia and negative urgency interaction. Gender and correlation paths have been omitted from the figure for clarity. All exogenous variables were allowed to freely correlate. The bulimia disturbance term was correlated with BulxNU and BulxDTS variables. All values are standardized coefficients. Standard errors are presented in parentheses. *p < .05, ** = *p* < .01, *** = *p *< .001

The hypothesized analysis first evaluated model fit [39]. The initial model was not an optimal fit to the data [*χ*^*2*^ (10, *N* = 429) = 47.8, *p* < .001; CFI = .97, RMSEA = .094 90% CI [.068–.121]. Modification indices were examined, and an additional path was added (negative urgency to body dissatisfaction). After this path was freed, the final model was a good fit to the data [*χ*^*2*^ (8, *N* = 429) = 25.69, *p* = .001; CFI = .98, RMSEA = .072 90% CI [.042–.104] (Fig. 1).

### Direct effects

As hypothesized, body dissatisfaction and negative urgency were positively associated with bulimia symptoms, while distress tolerance was inversely associated. Unexpectedly, male gender was associated with more bulimia symptoms. Lastly, negative urgency, negative affect, and female gender displayed direct effects to body dissatisfaction. R^2^ values and unstandardized coefficients are reported in (Fig. 1) and (Table 3), respectively.

**Table 3:** Unstandardized coefficients and standard errors of direct effects (*N* = 429)

Variable/path	Unstd. coef.	SE	*p*
*NSSI*			
EDI—bulimia	0.02*	0.01	.021
DTS	− 0.37***	0.07	< .001
Negative urgency	0.38***	0.11	< .001
EDI—bulimia-DTS	0.02**	0.01	.011
DTS-negative urgency	− 0.17	0.09	.065
EDI—bulimia-negative urgency	− 0.01	0.01	.508
Gender	− 0.03	0.11	.766
*R*^2^	0.45***	0.05	< .001
EDI—bulimia			
DTS	− 0.78*	0.38	.037
Negative urgency	4.12***	0.60	< .001
EDI—body dissatisfaction	2.39***	0.30	< .001
Gender	2.36***	0.61	< .001
*R*^2^	0.40***	0.04	< .001
EDI—body dissatisfaction			
Negative affect	0.24***	0.62	< .001
Negative urgency	0.34***	0.09	< .001
Gender	− 0.45***	1.00	< .001
*R*^2^	0.16***	0.03	< .001

EDI = eating disorder inventory, NSSI = non-suicidal self-injury, DTS = distress tolerance

Gender coded 1 (male) and 0 (female). NSSI is dichotomous (positive history = 1, no history = 0)

*p < 0.05, **p < 0.01, ***p < 0.001

**Fig. 2:**
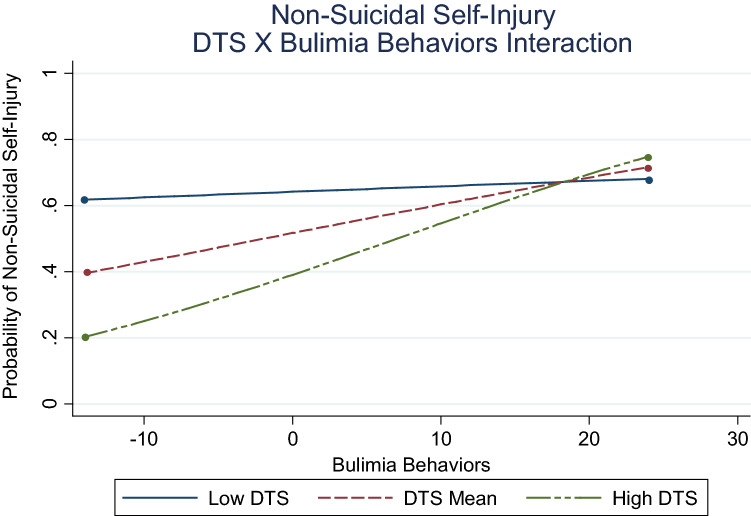
Associations between bulimia and NSSI as a function of distress tolerance. DTS = distress tolerance; low DTS = 1 SD below mean; high DTS = 1 SD above mean

Consistent with hypotheses, distress tolerance was inversely associated with NSSI. Bulimia symptoms and negative urgency were also positively associated with NSSI. The hypothesized interaction effect that distress tolerance would moderate the bulimia-NSSI association (Fig. 2) was supported (*β* = 0.02, *p* = .030), although the nature of the moderation was unexpected. Results indicated the association between bulimia symptoms and NSSI increased in magnitude from low (− 1 *SD; β* = 0.003, *p* = .916) to moderate (mean; *β* = 0.05, *p* = .004) to high (+ 1 *SD*; *β* = 0.12, *p* < .001) levels of distress tolerance. Notably, low distress tolerance consistently elevated likelihood of NSSI across varying levels of bulimia symptoms.

**Fig. 3:**
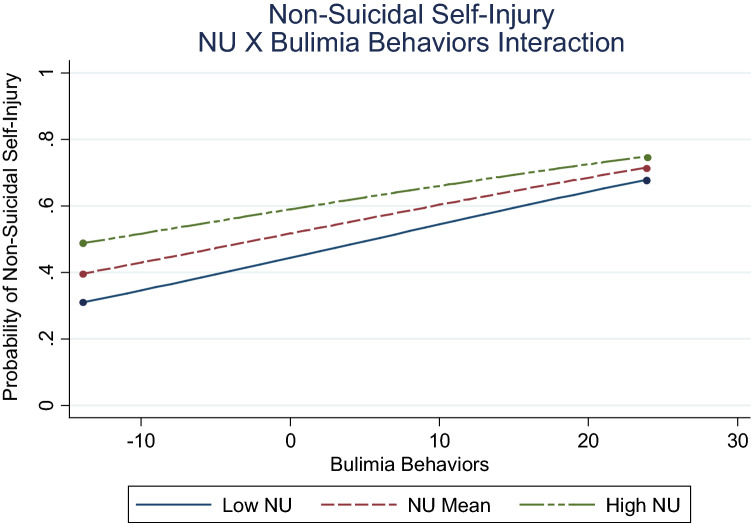
Associations between bulimia and NSSI as a function of negative urgency. NU = negative urgency; low NU = 1 SD below mean; high NU = 1 SD above mean

**Fig. 4:**
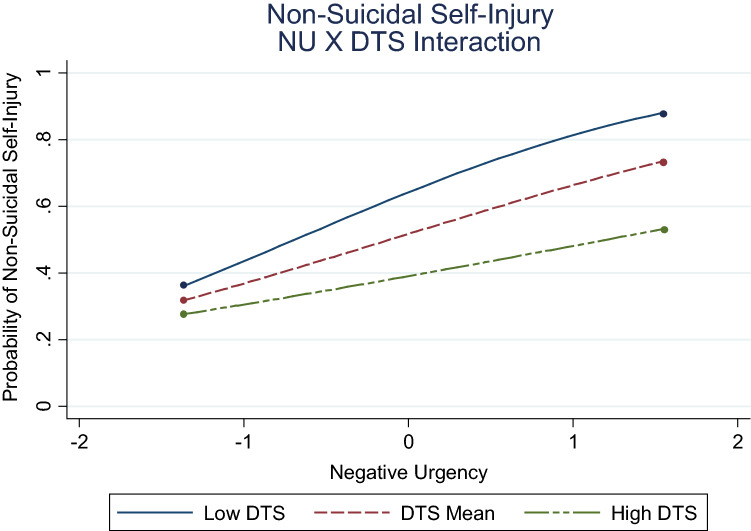
Associations between negative urgency and NSSI as a function of distress tolerance. NU = negative urgency; DTS = distress tolerance; low DTS = 1 SD below mean; high DTS = 1 SD above mean

The path of bulimia to NSSI was also conditional upon negative urgency (Fig. 3). Results indicated this association was attenuated from low (− 1 *SD; β* = 0.07, *p* = .021), to moderate (mean*; β* = 0.05, *p* = .004), to high (+ 1 *SD; β* = 0.04, *p* = .086) levels of negative urgency. Similarly, the nature of the distress tolerance-negative urgency interaction (Fig. 4) was attenuated from low (− 1 *SD; β* = 0.47, *p* = .046), to moderate (mean*; β* = 0.05, *p* = .004), to high (+ 1 *SD; β* = − 0.35, *p* = .118) levels of distress tolerance, consistent with hypothesis.

### Indirect effects

Indirect effects were calculated as the cross-products of coefficients. Significance of indirect effects was determined via bias-corrected bootstrapped confidence intervals, and 1000 samples were drawn and effects were estimated from each sample. Consistent with hypotheses, negative affect was associated with NSSI via body dissatisfaction and bulimia [*ab* = 0.012 95% Cl (0.001, 0.063)], and negative urgency was significantly associated with NSSI via body dissatisfaction and bulimia [*ab* = 0.018 95% Cl (0.001, 0.050)]. Moderated mediation of this indirect effect was significant, and a conditional indirect effect of negative urgency on NSSI via body dissatisfaction and bulimia was observed at low (− 1 *SD; β* = 0.02, *p* = .034), but not moderate (mean*; β* = 0.09, *p* = .331) or high (+ 1 *SD; β* = 0.07, *p* = .416) levels of negative urgency. This suggests body dissatisfaction and bulimia mediates the relationship between negative urgency and NSSI for those who display low levels of negative urgency. The hypothesized indirect effect of distress tolerance to NSSI via bulimia was not supported [*ab* = − 0.017 95% Cl (− 0.113, 0.005)].

## Discussion

This study examined specific configurations of emotion regulation in the relationship between bulimia symptoms and NSSI, accounting for nearly half of NSSI variance. To our knowledge, this is the first study to examine concurrent risk factors in comorbid presentations of bulimia symptoms and NSSI. Results also modeled the mediating role of bulimia symptoms between emotion regulation and NSSI, providing hypothesized temporal precedence of these outcomes. Consistent with previous findings [16], negative urgency and distress tolerance were significant vulnerability factors where the relationship between negative urgency and NSSI was strengthened at lower distress tolerance. Regardless of gender, greater deficits in emotion regulation place individuals at risk of developing dysregulated behaviors. Together, these effects lend support for the experiential avoidance model of self-harm behaviors and broaden the model to include eating pathology.

As expected, lower ability to tolerate distressing experiences, and greater impulsivity under negative affect were linked to greater likelihood of engaging in self-harm. These factors also moderated the relationship between bulimia and NSSI. Unexpectedly, moderate-to-high distress tolerance significantly strengthened this relationship. While this appears counter-intuitive, note that lower distress tolerance was associated with consistent elevated risk of NSSI engagement across varying bulimia symptoms. This suggests that individuals with low distress tolerance are at greater risk of engaging in self-harm behaviors, and the buffering effect of greater distress tolerance is meaningful at lower likelihood of self-harm and bulimia behaviors. As likelihood and engagement in these behaviors increase, the risk reduction associated with greater distress tolerance decreases. This suggests that reduced distress tolerance initially increases vulnerability to engage in maladaptive behaviors; however, increased engagement may be related to additional risk factors.

Indeed, the relationship between bulimia and self-harm strengthened as negative urgency increased. This is consistent with previous findings [2, 15, 40], and supports the role of emotion dysregulation among these outcomes. Notably, the risk promoting function of negative urgency strengthens this relationship at greater engagement of these behaviors. Inconsistent findings regarding direct effects of distress tolerance to NSSI have been observed, where effects were only significant when interacting with negative urgency [15, 40]. Together, these findings indicate increased vulnerability to initially engage in maladaptive behaviors in the presence of emotion regulation deficits; however, impulsivity may exert stronger influence at greater engagement in behaviors. These findings provide important clinical implications, as emotion regulation targets within treatment may vary given the intensity of behavioral engagement.

The finding that negative urgency displayed a positive relationship with body dissatisfaction is novel, and lends additional support for the experiential avoidance model among bulimia pathology. Individuals with greater impulsivity when faced with negative mood are more likely to engage in dysregulated eating behaviors or self-harm [41]. Following this engagement, cyclical patterns of guilt and shame develop [1, 21], and may be directed towards the self, resulting in greater experiences of body dissatisfaction. This reinforces engagement in dysregulated behaviors to reduce negative affective states.

Lastly, the finding that male gender was linked to bulimia is inconsistent with previous research [25]. This unexpected result may be an artifact of the sampling method. More male participants who endorsed lifetime-NSSI, were recruited, thus inflating the impact of gender on bulimia symptoms. However, results from Kwan and colleagues’ [42] prospective study suggest bulimia behaviors increased over time among college-aged men. Results from the current study may reflect this phenomenon. Further research, employing prospective studies at the within-person level is needed to clarify this relationship.

There are considerable strengths of the current study, including the depth and complexity of results following a sophisticated analysis. Results integrate a number of risk factors and support the hypothesized experiential avoidance model as it relates to comorbid presentations of bulimia symptoms and self-harm. However, the current study is not without limitations. First, data collected may have been vulnerable to reporter bias given the use of self-report measures. Future research could implement behavioral paradigms to further investigate the impact of these factors within this relationship.

Second, as self-injury was an inclusion criterion for MTurk participants, differences across recruitment sources were unable to be tested. However, we were interested in a high-risk sample, which was obtained through this recruitment strategy. Although the sampling method provided a considerable proportion of participants with a history of eating pathology and NSSI, prevalence rates in a community, young adult sample cannot be inferred. Finally, causal inferences cannot be made given the cross-sectional, non-experimental design. The directions of the paths are theoretical in nature as data were collected at one point in time. In an effort to establish causal inferences, further research employing prospective and experience sampling designs is needed. However, the current study extends etiological models of self-harm to include eating pathology, and highlights unique contributions of emotion regulation across comorbid presentations.
